# Editorial: Ovarian aging and reproduction

**DOI:** 10.3389/fendo.2022.1081348

**Published:** 2022-11-28

**Authors:** Mengyu Liu, Dan Zhang, Wenzhu Li, Bufang Xu, Huai L. Feng

**Affiliations:** ^1^ Reproductive Medical Center, Department of Obstetrics and Gynecology, Ruijin Hospital, Shanghai Jiao Tong University School of Medicine, Shanghai, China; ^2^ New York Fertility Center, New York-Prebyterian Healthcare System Affiliate Weill Cornell Medical College, New York, NY, United States

**Keywords:** ovarian, aging, art, POR, POI

## Introduction

Women’s reproductive ability declines from the early 30s, with the decline accelerating around the mid-30s. In women in their 40s, half of all infertility is closely related to ovarian aging (1, 2). With the postponement of childbearing and the adjustment of population structure in modern society, ovarian aging and its related problems are becoming more serious, such as the risk of infertility and pregnancy-related complications (3). Meanwhile, the asynchronous aging of ovaries and the extension of women’s life span has resulted in damage to the physical and mental health of advanced-age women. Recent studies demonstrated that many factors affect ovarian aging and consequently impact ovarian function and reproductive ability.

## The main points from previous publications

This topic includes thirty papers: four review articles, three systematic reviews, one clinical research, and twenty-two original research articles. In this editorial, the relevant contents are summarized as follows.

### Molecular mechanisms related to ovarian function and ovarian aging

Ovarian aging is principally determined by the quantity and quality of follicles, which are the functional units of the ovary. Ovarian follicle development is regulated by complex signaling pathways and steroid hormones. Li et al. comprehensively summarized and compared the roles of phosphatidylinositol-4,5-bisphosphate 3-kinase (PI3K)/protein kinase B (AKT)/forkheadbox O3 (FOXO3), wingless-related integration site (WNT), insulin, Notch, and Hedgehog signaling pathways in ovarian follicle development, and found that the above pathways all interacted with FOXO3, a transcription factor that suppresses the over-activation of follicles at the early stage and contributes to recruitment from primordial follicles. Besides, those signaling pathways and hormones synergistically participate in follicle development. Estrogen and estrogen receptors (ERs) play an important role in estrogen signal transduction and regulating the normal physiological function in ovaries. Xu et al. elucidated the underlying pathological mechanism between ERs (Erα and ERβ) and certain ovarian diseases, such as polycystic ovary syndrome (PCOS), ovarian cancer, and premature ovarian failure (POF), providing a potential therapeutic direction for ovarian dysfunction.

Oocytes contain the largely abundant mitochondria that produce ATP to support fertilization ability and early embryo development. Healthy mitochondria are important for female reproduction, as proven by the fact that in ovarian aging, the progressive impairment of mitochondrial activities is observed in both ovarian germ and somatic cells, which contributes to the imbalance of redox and metabolic alterations. The communication between mitochondria and the nucleus has been extensively characterized. Colella et al. described the role of mitochondria non-coding RNA (ncRNA, i.e., mitochondrial microRNAs (mitomiRs) and mitochondrial long noncoding RNAs (mtlncRNAs)) in nuclear-mitochondrial crosstalk in ovary aging and the regulatory mechanism exerted by the pituitary-ovarian axis. It has been hypothesized that mitochondria in granulosa cells change during aging, which could negatively affect oocyte quality and function. Hence, Alberico and Woods investigated the underlying molecular mechanism between mitochondrial aging in granulosa cells and the subsequent decline in fertility in female mammals. Additionally, Qin et al. compared the different gene expression profiles between *in vitro* maturation (IVM) and *in vivo* maturation (IVO) oocytes from aged mice and found that oxidative phosphorylation (OXPHOS) was the most enriched pathway, which is an important metabolic pathway in mitochondria.

A previous study identified the alterative gut microbiota of patients with ovarian dysfunction, especially PCOS (4). However, the alterations in the blood microbiota of PCOS were unclear. In this section, Wang et al. investigated the microbiome profile of PCOS using 16S RNA sequencing compared with healthy control women and found that there was lower alpha diversity, different beta diversity, and many taxonomic variations in the PCOS group. Besides, the relationship between gut microbiota and premature ovarian insufficiency (POI) also remains to be determined. Jiang et al. found that in patients with POI, the abundance of the genus *Eggerthella* was increased in feces, and the level of transforming growth factor beta 1 (TGF-β1) was increased in the serum. Moreover, the above two indicators were reversed in POI patients who received hormone replacement therapy (HRT) suggesting that the interactions among gut microbiota, serum metabolites, and TGF-β1 might participate in the development of POI.

### Predictive factors that affect ovarian reserve and reproductive outcomes

There is an urgent need to identify predictive factors associated with reproductive outcomes to guide clinical therapy in assisted reproductive technology (ART), not only to reduce clinical expense but also to obtain a higher pregnancy rate and better-personalized treatment. Firstly, age was the most important factor that affects ovarian function and reproductive outcomes. In a retrospective study, Liu et al. analyzed the cumulative live birth rate (CLBR) in different advanced-age women (38–39, 40–41, 42–43, and older than 43 years) who received a single ovulation induction cycle and explored the possible influencing factors. The results showed that the critical cutoff of achieving reasonable CLBR in women with advanced age after single stimulation might be 42 years.

Besides, in recent years, Anti-Müllerian hormone (AMH) and antral follicle count (AFC) have been widely used for ovarian reserve assessment. Sun et al. enrolled 2585 infertile women to evaluate the predictive ability of AFC and AMH for ovarian response in the *in vitro* fertilization/intracytoplasmic sperm injection (IVF/ICSI) process, which showed that the numbers of retrieved oocytes correlated positively with both factors. AFC and AMH had comparable efficacies (area under the curve (AUC) = 0.731 and AUC = 0.733, respectively) in high ovarian response prediction. However, AMH and AFC might vary across different ethnic populations; therefore, Melado et al. described the landscape of AMH and AFC in 2495 women aged 19 to 50 years who were native to the Arabian Peninsula, providing an increased understanding of ovarian reserve prediction. In addition, in another study in this series, Wu et al. found that there was no significant correlation between ovarian reserve function and thyroid function in infertile women.

Poor ovarian response (POR) is an intractable clinical challenge in ART, leading to a higher cycle cancellation rate and lower pregnancy rate. It is necessary to explore the possible predictive factors for POR to direct clinical decisions. Based on this, Li et al. developed a nomogram to predict clinical pregnancy failure during IVF/ICSI, which found that age older than 35 years, body mass index (BMI) >24 kg/m^2^, type B or C of endometrium on Trigger day, basic follicle-stimulating hormone (FSH)>10 mIU/ml, basic E_2_>60 pg/ml, and less than two high-quality embryos were closely associated with pregnancy failure in patients with PORs. Similarly, in patients with unexpected sub-optimal response during ovulation induction, Wang et al. presented age, BMI, and basal follicle-stimulating hormone (FSH) were independent risk factors, while the initial dosage of gonadotropin (Gn), FSH, and luteinizing hormone (LH) on the first day of Gn were independent protective factors for a sub-optimal response.

Smooth endoplasmic reticulum clusters (SERCs) are an organelle for the storage and redistribution of calcium in oocytes, which is responsible for cell activation (5). Currently, it is still controversial as to whether SERCs in oocytes could affect the development of blastocysts or even reproductive outcomes. In a retrospective study, Wang et al. found that SERCs might adversely affect the quality and speed of blastocyst development, such as the fertilization rate, good-quality blastocyst rate, and the proportion of trophectoderm grade C in blastocysts.

## New strategies for enhancing ovarian function and improving pregnancy outcomes

### New advances in the therapy of POI

With the development of medical technology and increasing affluence, patients with POI have an increasingly strong desire to become pregnant. Patients with POI have intermittent ovarian functions and 5–10% of POI patients have a chance to conceive (6). Ishizuka et al. described the live birth rate in patients with POI during hormone replacement therapy with or without ovarian stimulation in a retrospective cohort study. The results indicated that infertility therapy was feasible in some patients with POI, especially the following two types of patients: a. Patients with idiopathic POI who initiated HRT before they were 35 years old; b. Patients who had undergone surgical treatment for benign ovarian tumors and the time of amenorrhea was less than 4 years.

In recent years, how to improve the fertility of patients with POR/POI has become a challenge and hot topic in the field of reproduction. With the development of regenerative medicine, a variety of bioactive substances have been reported to be able to improve ovarian function, such as stem cells, growth hormones, and dehydroepiandrosterone. Liu et al. acquired a cell-free fat extract (CEFFE) from adipose tissue that contained more than 1000 protein components and could effectively improve ovarian function and fertility in mice with POI or advanced age (7). In addition, other studies suggested that pyrroloquinoline-quinine (PQQ) and human umbilical cord mesenchymal stem cell-derived exosomes (hUCMSC-Exos) could improve the ovarian function in POI mice induced using an alkylating agent, including the increased follicle number and recovered sex hormone levels (Dai et al. and Li et al.).

### New advances in the therapy of POR

In a previous study, the growth hormone (GH) and GnRH were co-applied in patients with POR according to the POSEIDON criteria. The data showed that GH cotreatment improved the live birth rate and clinical pregnancy rate in all four groups (Liu et al.). Wu et al. retrospectively evaluated the efficacy between IVF and intrauterine insemination (IUI) in patients with unexplained infertility of POSEIDON group 3. They found that the live birth rate per IVF cycle was higher than the cumulative live birth rate of IUI. Zhang et al. conducted a retrospective cohort analysis on 67 patients with unpredictable POR undergoing IVF/ICSI therapy to examine the effect of early (stimulation day 1) and mid-late (stimulation day 6) follicular phase administration of 150 IU of human chorionic gonadotropin (hCG) in the subsequent gonadotropin hormone-releasing hormone (GnRH) antagonist cycle. The data showed that adding 150IU of hCG at the early follicular phase significantly increased the number of oocytes retrieved, the maturity of the oocytes, and the number of usable embryos than at the mid-late phase.

### New advances in anti-aging of the ovary

Over the past 150 years, women’s life expectancy has increased from 45 years to 85 years. However, reproductive aging remained constant at 50-52 years (8). Nowadays, more women choose to postpone childbearing. Hence, it is necessary to explore effective inventions to prevent the senescence of the ovaries. Some studies have reported that antioxygen could delay ovarian aging. Among them, coenzyme (CoQ10) plays a critical role in mitochondrial metabolism, antioxidant, and anti-aging, and could improve oocyte quality and delay oocyte senescence (9). Based on network pharmacology, Yang et al. explored the underlying mechanism of CoQ10’s anti-aging effect on oocytes and identified some vital pathways such as peroxisome proliferator-activated receptor (PPAR), tumor necrosis factor (TNF), and mitogen-activated protein kinase (MAPK) signaling pathways, and seven hub genes (e.g.,*PPARA* (encoding peroxisome proliferator-activated receptor A), *CAT* (encoding catalase), and *MAPK14* (encoding mitogen-activated protein kinase 14)), which might be involved in the mechanism. Besides, Pando et al. found that deficiency of NLR family pyrin domain containing 3 (NLPR3) or an NLPR3 inhibitor could both effectively improve the ovarian function and fertility of mice by alleviating ovarian inflammation, which provided a new therapeutic target for anti-aging of the ovary (10). Another study showed that a cell-free fat extract could improve the ovarian function of advanced-age mice by resisting the DNA damage of granulosa cells (11). Miao et al. also demonstrated that mononucleotide (NMN) supplementation could effectively protect oocytes from functional decline with age and eventually improved the fertility of advanced-age mice (12).

### Spindle transfer technique for aging oocytes

Mutations of mitochondrial DNA (mtDNA) could be inherited from the mother to her offspring. Currently, there are limited methods to prevent the transfer of mutated mtDNA. Oocyte reconstruction by spindle transfer (ST) is a feasible strategy for mitochondrial function-impaired diseases and aging oocytes. However, the efficiency of ST is limited because of the chromosome abnormality in the reconstructed oocytes (13). Wang et al. found that electrofusion stimulation was an independent factor for chromosome abnormality during ST by decreasing maturation-promoting factor (MPF) activity, leading to premature chromosome separation and abnormal spindle morphology, which was not related to enucleation, fusion status, temperature, and Ca^2+^.

## Synthesis and conclusion

Taken together, the above studies published on this Research Topic provide novel factors affecting ovarian function, which could lead to a better comprehension of the mechanism underlying ovary aging. Meanwhile, this topic also covers new strategies to improve ovarian function and reproductive outcomes from both biological and clinical aspects, which might provide new insights into treatment options.

## Author contributions

ML drafted this editorial. DZ and WL contributed to the literature survey. BX and HF revised and modified the editorial. All authors contributed to the article and approved the submitted version.

## Funding

This work was supported by grants from the National Natural Science Foundation of China (No.82071712, No.81873857, No.82071596, and No.82101800), the Guangci Clinical New Technology Sailing Plan of Ruijin Hospital (GCQH-2021-07), the Shanghai Medical and Health Development Fund “Yiyuan Star Outstanding Young Physician” Project (SHWJRS(2021)-99), and the Beijing Health Promotion Association’s “Reproductive Medicine Young and Middle-aged Clinical Research” Project (BJHPA-2022-SHZHYXZHQNYJ-LCH-009).

## Conflict of interest

The authors declare that the research was conducted in the absence of any commercial or financial relationships that could be construed as a potential conflict of interest.

## Publisher’s note

All claims expressed in this article are solely those of the authors and do not necessarily represent those of their affiliated organizations, or those of the publisher, the editors and the reviewers. Any product that may be evaluated in this article, or claim that may be made by its manufacturer, is not guaranteed or endorsed by the publisher.
